# Sensor-based evaluation of intermittent fasting regimes: a machine learning and statistical approach

**DOI:** 10.1038/s41366-025-01889-0

**Published:** 2025-08-22

**Authors:** Nico Steckhan, Tanja Manlik, Tillmann Int-Veen, Beeke Peters, Christina Laetitia Pappe, Daniela A. Koppold, Bert Arnrich, Andreas Michalsen, Henrik Dommisch, Peter Schwarz, Olga Pivovarova-Ramich

**Affiliations:** 1 Evidence-based Digital Diabetology, Department of Medicine III, Faculty of Medicine Carl Gustav Carus, Technische Universität Dresden, Dresden, Germany; 2 Digital Health—Connected Healthcare, Hasso Plattner Institute, University of Potsdam, Potsdam, Germany; 3 Department of Molecular Metabolism and Precision Nutrition, German Institute of Human Nutrition Potsdam-Rehbruecke, Nuthetal, Germany; 4 German Center for Diabetes Research (DZD), München-Neuherberg, Germany; 5 Research Group Molecular Nutritional Medicine and Department of Human Nutrition, German Institute of Human Nutrition Potsdam-Rehbruecke, Nuthetal, Germany; 6 Department of Periodontology, Oral Medicine and Oral Surgery, Charité—Universitätsmedizin Berlin, Corporate Member of Freie Universität Berlin, Humboldt-Universität zu Berlin, and Berlin Institute of Health, Berlin, Germany; 7 Department of Internal and Integrative Medicine, Immanuel Hospital Berlin, Berlin, Germany; 8 Institute of Social Medicine, Epidemiology and Health Economics, Charité—Universitätsmedizin Berlin, Corporate Member of Freie Universität Berlin, Humboldt-Universität zu Berlin, and Berlin Institute of Health, Berlin, Germany; 9 Department of Periodontology, Health Science Center, University of Washington, Seattle, WA USA; 10 Charité—Universitätsmedizin Berlin, Corporate Member of Freie Universität Berlin, and Humboldt-Universität zu Berlin, Department of Endocrinology and Metabolism, Berlin, Germany

**Keywords:** Nutrition, Translational research

## Abstract

The primary aim was to develop and assess the performance and applicability of different models utilizing sensor data to determine dietary adherence, specifically within the context of intermittent fasting. Our approach utilized time-series data from two completed human trials, which included continuous glucose monitoring, acceleration data, and food diaries, and a synthetic data set. Machine learning models achieved an average F1-score of 0.88 in distinguishing between fasting and non-fasting times, indicating a high level of reliability in classifying fasting states. The Hutchison Heuristic statistical method, while more moderate in performance, proved to be robust across different cohorts, including individuals with and without type 1 diabetes. A dashboard was developed to visualize results efficiently and in a user-friendly manner. The findings highlight the effectiveness of using sensor data, combined with advanced statistical and machine learning approaches, to passively evaluate dietary adherence in an intermittent fasting context.

## Introduction

In clinical research, the data interpretation relies heavily on study compliance, meaning the extent to which participants adhere to the study protocol [1]. This is crucial in human studies where self-reporting over extended periods is required.

Common methods for measuring compliance include clinician impressions, patient interviews, prescription records, monitoring devices, and pharmacological markers. Patient interviews are straightforward and cost-effective, while pharmacological markers provide objective assessments but require ethical consideration [2].

Fasting studies often monitor ketone levels from urine, blood, or use newly developed breath ketone monitors to objectively measure adherence, but these methods require active participation and ketone increase is under the detection limit upon short fasting duration such as 16:8 fasting. Conversely, video surveillance or clinic-based meal consumption methods lack blinding [3]. Alternatively, participants may use photo and text food diaries [4, 5] or log fasting times to track adherence.

Mobile and wearable technologies, including accelerometers, glucose monitors, and heart rate monitors, are increasingly used to monitor health-related parameters. Type 2 diabetes, the most common and preventable form of diabetes, is frequently studied [6]. Despite challenges in wearable technologies for dietary intake, such as transient signal loss and inaccurate calorie estimations [7], recent advancements in mobile sensors have improved the timeliness of dietary information. Continuous glucose monitoring (CGM) and activity tracking enable passive, unobtrusive data collection, enhancing compliance, reducing participant burden, and generating vast amounts of dynamic data, which are well-suited for advanced machine learning applications. Integrating data and machine learning in clinical research can predict diabetes risk and detect early signals of glucose metabolism disorders [8–10].

Therefore, the primary aim of this study was to develop and assess the performance and applicability of different models utilizing sensor data to determine dietary adherence, e.g. the adherence of eating duration and the timely adherence regarding specific time-frames for eating within the context of intermittent fasting.

## Materials and methods

### Data collection

**Fig. 1:**
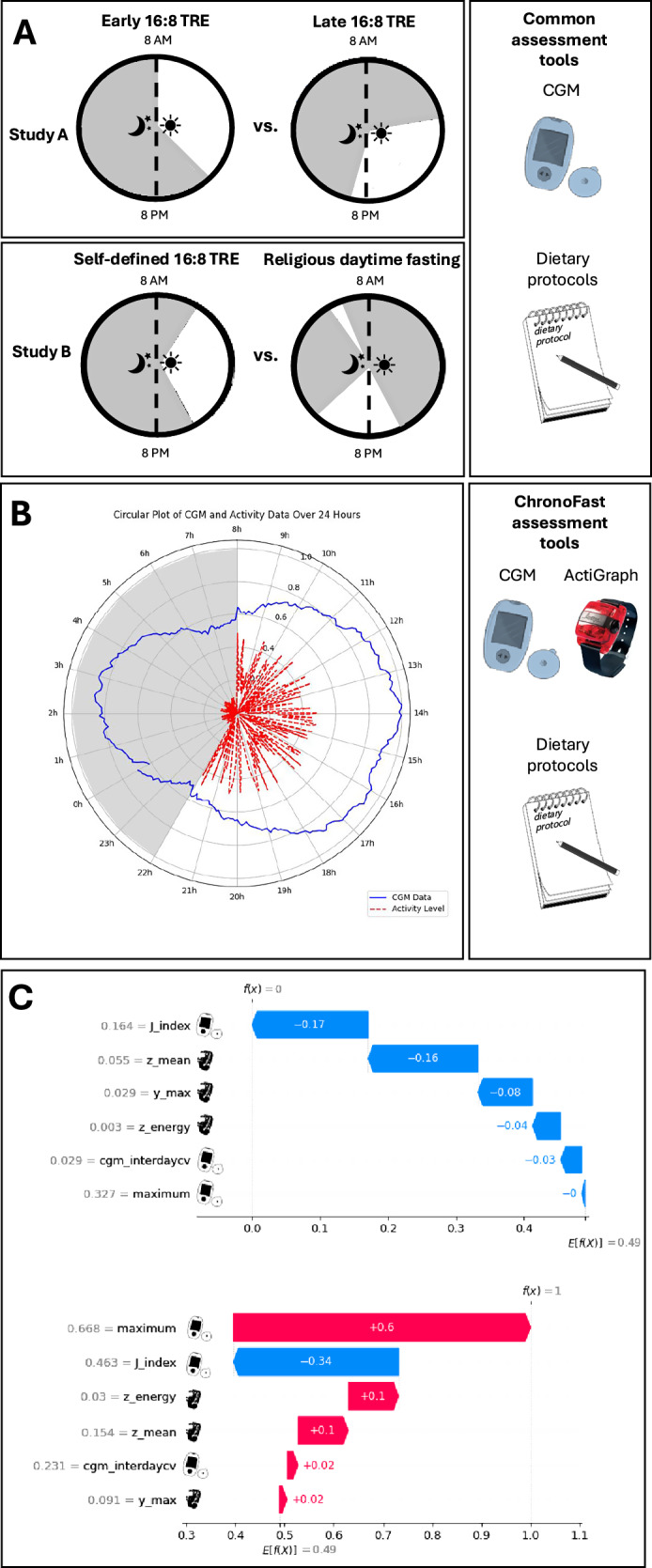
Designs, data, and machine learning interpretation of fasting states. **A** Designs and circadian profiles of study A (ChronoFast) and study B (ParoFastIN). **B** Sensor data of an average day of a representative person showing circadian differences at day and night (simulated normalized data, blue line: glucose level, red line: activity level). **C** SHAP (Shapley Additive exPlanations) values for local interpretation of a sample predicted as “fasting” (f(x) = 0) and “non-fasting” (f(x) = 1). The *X*-axis represents the SHAP values, indicating the degree of change in log odds. The *Y*-axis lists the feature names. Red arrows indicate positive feature attribution (increasing the log odds), while blue arrows indicate negative feature attribution (decreasing the log odds). The baseline prediction is E[f(X)] = 0.49, representing the model’s average prediction across the dataset. intradaycv CV (Coefficient of Variation) is calculated by 100 ∗ sd(G)/mean(G) of all days in data, j index j index is a measure of quality of glycemic control based on the combination of information from the mean and SD calculated as 0.001 × (mean + SD), maximum maximum glucose value, y_max maximum acceleration along *y* axis, z_energy energy of movement along the Z-axis, z_mean mean (average) acceleration along the *Z*-axis [CGM continuous glucose monitoring, TRE time-restricted eating]. Parts of the figure were drawn using Servier Medical Art licensed under CC BY 4.0 (https://creativecommons.org/licenses/by/4.0/).

Data was collected in two human trials on different intermittent fasting regimes, in which designs, eligibility criteria, and ethic approvals were published elsewhere [11, 12]. In brief, the cross-over ChronoFast trial (ClinicalTrials: NCT04351672) investigated the effect of early (eating 08:00 to 16:00 h) versus late (eating 13:00 to 21:00 h) time-restricted eating (TRE) on glucose values collected for 14 days during each intervention and baseline in 31 women [11]. The three-arm ParoFastin trial (DKRS: DRKS00026701) studied the impact of TRE (eating within a self-selected 8-h period) versus religious dry fasting (eating before sunrise and after sunset), versus a habitual diet on glucose values collected for 19 days during each intervention and 3–7 days of baseline in 16 subjects (Fig. 1A). Parallel with glucose data, food logs were collected in both trials, whereas acceleration data (physical activity) were assessed in the ChronoFast trial only (Fig. 1B). Written informed consent was obtained from all subjects.

### Data description

Acceleration data in ChronoFast trial were assessed using an wGT3X-BT sensor (ActiGraph, USA) on participants’ non-dominant wrists. Data was extracted after baseline and intervention periods (eTRE and lTRE) using ActiLife software (ActiGraph, USA), requiring participants’ height, weight, age, and sex. The outputted .agd file (1 Hz): timestamp, participant ID, *x*-, *y*-, *z*-axis acceleration, steps, lux levels, vector magnitude, and times per minute spent standing, sitting, lying, or having the actigraph off.

Glucose data were collected by a FreeStyle Libre 2 sensor in the ChronoFast trial and a FreeStyle Libre Pro IQ sensor in the ParoFastin trial (both from Abbott, Wiesbaden, Germany). Both sensors performed a CGM saving the glucose readings every 15 min, while the FreeStyle Libre 2 sensor have to be scanned with a reading device at least every 8 h. Data was extracted after each period using FreeStyle Libre software (Abbott, Wiesbaden, Germany) as CSV files.

Nutrition data were collected by participants after the initial advise by a nutritionist using the Fddb Extender app (Food Database GmbH) or paper-based records, which were transferred into the Fddb app for the primary nutritional analysis [13]. Data were exported into Excel files, detailing meal times as well as daily eating and fasting periods. The adherence to fasting regimens was assessed based on the eating duration of 8 h (for both ChronoFast and ParoFastin trials) and to prescribed time-frames (for the ChronoFast trial only). Days at which subjects followed the dictated 8 h windows ± 30 min were counted as compliant [12].

Further, we utilized Simglucose, a Python package designed for simulating T1DM time series data. Simglucose allows for various configurations, such as selecting the age of simulated individuals and the glucose measurement device. Since managing T1DM requires insulin administration, the simulation incorporates insulin delivery via an insulin pump following each meal (Supplementary B).

### Data preparation

Data were analyzed in Python 3.9 using Jupyter Notebook and PyCharm. All three data sources, available in JSON and Excel/CSV formats, were imported into data frames using the pandas library. For data analysis, these sources were harmonized into a single data instance. Specifically, the sampling rate of the acceleration data was downsampled to match the sampling rate of the glucose data. Nutrition data was incorporated as a categorical variable labeled “fasting” or “non-fasting.”

### Machine learning

Using data from the ChronoFast study, we compared various approaches to develop a precise and stable binary classification predictor of fasting state based on time series data:

1. Hutchison heuristic: mean fasting glucose for a participant in a given condition (baseline, TRE early, and TRE delayed) over a “fasting” window of 4 h after the latest meal and across all days. The “fed” window is from the earliest eating occasion until the latest meal occasion, plus 4 h to allow for postprandial fluctuations [14].
2. Supervised machine learning: several machine learning models are trained on features from overnight fasting data and patient-reported outcomes.
3. Supervised time series classification: classification models trained on raw data and on features from overnight fasting data and patient-reported outcome.

The resulting models were evaluated in ParoFastin dataset and simulated data of type 1 diabetes by the package simglucose [15].

#### Feature engineering and selection

The first feature computation was done on the glucose data with the use of the cgmquantify Python package [16]. Twenty-four metrics of glycemic control and variability were computed. The second feature computation was performed on the acceleration data. The acceleration features were mainly computed based on methods published in a paper about activity recognition [17]. The scipy.signal package [18] was used as a tool for calculations and for the identification of signal peaks. Both feature computation steps were computed over a time window of 45 min for the training part.

To reduce model complexity and improve performance, feature selection was conducted to identify most relevant variables using Recursive Feature Elimination (RFE) from the scikit-learn package [19]. Computed features are presented in Table 1.

**Table 1:** List of computed features from multimodal sensors.

Data source	Computed features
Glucose	Mean glucose
Glucose	Mean of Interdaycv (of 45 min feature)
Glucose	Mean of J-index (of 45 min feature)
Glucose	Mean of glucose maximum (of 45 min feature)
Acceleration	Mean steps
Acceleration	Mean vector magnitude
Acceleration	Mean of *z*-axis mean (of 45 min feature)
Acceleration	Mean of *y*-axis max (of 45 min feature)
Acceleration	and mean of *z*-axis energy (of 45 min feature)

#### Model approach

Balancing and restructuring the dataset was conducted to prevent overrepresentation of any label, thus avoiding overfitting. Techniques from the Imbalanced-learn package [20] were used for this purpose. For classification tasks, SMOTETomek [21] was applied, combining over- and undersampling to maintain data quality. SMOTE [22] generated new samples by interpolating between marginal outliers and inliers, while cleaning methods such as nearest neighbors reduced noise. This approach retained the dominant label class without excessive noise. For time series classification, a Random Undersampler addressed varying series lengths.

Post-balancing, features were scaled using the Min-Max scaler from scikit-learn [19], normalizing data to a range between 0 and 1. This scaling was crucial for handling skewed data and outliers, improving algorithm accuracy and reducing time complexity. Various supervised machine learning models were then tested. SHapley Additive exPlanations (SHAP) values were computed to achieve global interpretability of the models (Fig. 1C).

### Dashboard

To provide robust data visualization, an easy-to-use interface with configurable options was developed using Dash library, designed to run on multiple computer instances. Visualizations were implemented with Plotly (Supplementary C).

## Results

Supervised machine learning algorithms were evaluated to compare the performance with which someone’s fasting state can be predicted (Table 2). The evaluation took place on the ChronoFast study (with train-test split), the ParoFastin study and a simulated type 1 diabetes data set. Combined with the simulated diabetes data set, all data sets are distinct from one another and contain individuals who, while similar in one cohort, exhibit different metabolic characteristics outside of the cohort.

**Table 2:** Performance scores of the fasting state prediction applied on the test data set of the ChronoFast study.

Prediction method	Data source	Accuracy	Smoothed accuracy	Precision	Recall	F1-score 95% CI
Support vector machine	Glucose, Acceleration	83%	87%	0.86	0.9	0.88 (0.87–0.89)
Multi-layer perceptron	Glucose, Acceleration	79%	84%	0.83	0.86	0.84 (0.81–0.85)
Random Forest	Glucose, Acceleration	79%	84%	0.8	0.91	0.85 (0.84–0.86)
K-nearest neighbors	Glucose, Acceleration	77%	82%	0.8	0.85	0.82 (0.81–0.83)
Time series specific K-nearest neighbors	Glucose, Acceleration	71%	82%	0.76	0.42	0.54 (0.53–0.54)
Decision Tree	Glucose, Acceleration	77%	82%	0.8	0.86	0.83 (0.81–0.83)
Logistic Regression	Glucose, Acceleration	77%	81%	0.81	0.81	0.81 (0.81–0.82)
Stochastic gradient descent	Glucose, Acceleration	77%	79%	0.8	0.8	0.8 (0.79–0.81)
Time series specific K-nearest neighbors (Raw)	Glucose, Acceleration	68%	79%	–	–	–
Time series specific Support Vector Classifier (Raw)	Glucose, Acceleration	75%	77%	–	–	–
Time series specific Support Vector Classifier	Glucose, Acceleration	69%	73%	0.69	0.63	0.7 (0.7–0.71)
Hutchison Heuristic	Glucose, Nutrition	67%	68%	–	–	–

The table is sorted by smoothed accuracy, “Raw” indicates a variant that the model was built from raw data and not from preprocessed features.

*CI* confidence interval.

The ChronoFast study engineered features from glucose, acceleration (Table S1), and fasting state labels using a sliding window technique, creating approximately 55,000 45-min windows. Recursive feature elimination identified three critical glucose features and three acceleration features for model training (Fig. 1C).

The best-performing models were the Support Vector Machine, the Random Forest, and the Multi-layer Perceptron, with f1-scores of 0.88, 0.85, and 0.84, respectively. Time series classification models initially performed worse but improved significantly with the application of a smoothing function. The Hutchison prediction method was the least effective, suggesting that acceleration data is crucial for more accurate predictions.

## Discussion

The introduced methods for predicting fasting states demonstrated their effectiveness in distinguishing fasting from non-fasting conditions. Within the ChronoFast training cohort, a maximum f1-score of 0.88 was achieved. Validation in other cohorts and fasting paradigms yielded a maximum accuracy of 73%, supporting the hypothesis that fasting state models can be generalized to a certain degree using sensors. Blood markers like ketones are the gold standard method to assess fasting states [23]. Prior research indicates that body parameters such as glucose and liquid profiles can differentiate fasting from non-fasting states [14, 24].

The study employed the Hutchison method, which uses mean fasting glucose as a personalized threshold value, to create a compliance method. The study highlighted the inadequacy of threshold methods like Hutchison’s heuristic for diabetes Type-1 patients due to the influence of insulin interventions. This inspired the incorporation of additional features from glucose and acceleration data into machine learning models. The resulting dashboard visualizes fasting state predictions, similar to existing CGM evaluation tools like the CGM Discussion viewer [25]. Unlike previous studies where acceleration data slightly improved glucose predictions [26], this study found significant accuracy improvements when combining acceleration with glucose data.

This research represents the first application of machine learning for dietary compliance evaluation in nutritional studies based on sensor data, offering an objective measure over traditional methods like video surveillance. However, this study relied on the assumption of overnight fasting. Further the use of ketones levels could enable more robust models for fasting state prediction.

Future directions include training models with more diverse data from different fasting regimens (e.g., 2-day diet, Every-Other-Day Diet) and including participants across a broad spectrum of health conditions, ages, ethnicities, and genders. Investigating a broader selection of machine learning models, such as long short-term memory models and recurrent neural networks, could further enhance accuracy [26]. Real-time compliance checks using AI dashboards, similar to those used for drug level adherence [27], could be adapted for dietary compliance, enabling rapid detection and intervention.

## Conclusion

The findings highlight the effectiveness of using glucose and activity sensor data, combined with advanced statistical and machine learning approaches, to passively evaluate dietary adherence without the burden of writing food diaries. Additionally, a novel dashboard for visualizing predictions was implemented. This approach can be utilized in clinical nutritional trials and field research.

## Supplementary information

Table S1: Summary table of the included study.

## Data Availability

https://github.com/xuantanja/ChronoFast-Dashboard.

## References

[1] Besch CL. Compliance in clinical trials. AIDS. 1995;9:1–10. 7893432 10.1097/00002030-199501000-00001

[2] Czobor P, Skolnick P. The secrets of a successful clinical trial: compliance, compliance, and compliance. Mol Interv. 2011;11:107–10. 21540470 10.1124/mi.11.2.8 PMC3109858

[3] Sutton EF, Beyl R, Early KS, Cefalu WT, Ravussin E, Peterson CM. Early time-restricted feeding improves insulin sensitivity, blood pressure, and oxidative stress even without weight loss in men with prediabetes. Cell Metab. 2018;27:1212–21.e3. 29754952 10.1016/j.cmet.2018.04.010 PMC5990470

[4] Wilkinson MJ, Manoogian ENC, Zadourian A, Lo H, Fakhouri S, Shoghi A, et al. Ten-hour time-restricted eating reduces weight, blood pressure, and atherogenic lipids in patients with metabolic syndrome. Cell Metab. 2020;31:92–104.e5. 31813824 10.1016/j.cmet.2019.11.004 PMC6953486

[5] Sahoo D, Hao W, Ke S, Xiongwei W, Le H, Achananuparp P, et al. FoodAI: food image recognition via deep learning for smart food logging. In: Proceedings of the 25th ACM SIGKDD international conference on knowledge discovery & data mining. Anchorage AK USA: ACM; 2019. pp. 2260–8. 10.1145/3292500.3330734

[6] Rodriguez-León C, Villalonga C, Munoz-Torres M, Ruiz JR, Banos O. Mobile and wearable technology for the monitoring of diabetes-related parameters: systematic review. JMIR MHealth UHealth. 2021;9:e25138 . 34081010 10.2196/25138 PMC8212630

[7] Dimitratos SM, German JB, Schaefer SE. Wearable technology to quantify the nutritional intake of adults: validation study. JMIR MHealth UHealth. 2020;8:e16405. 32706729 10.2196/16405 PMC7407252

[8] García-Ordás MT, Benavides C, Benítez-Andrades JA, Alaiz-Moretón H, García-Rodríguez I. Diabetes detection using deep learning techniques with oversampling and feature augmentation. Comput Methods Prog Biomed. 2021;202:105968. 10.1016/j.cmpb.2021.105968 33631638

[9] Tripathi G, Kumar R. Early prediction of diabetes mellitus using machine learning. In: 2020 8th international conference on reliability, infocom technologies and optimization (trends and future directions) (ICRITO). Amity University, Noida, India. 2020. pp. 1009–14.

[10] Naz H, Ahuja S. Deep learning approach for diabetes prediction using PIMA Indian dataset. J Diab Metab Disord. 2020;19:391–403. 10.1007/s40200-020-00520-5 PMC7270283 32550190

[11] Peters B, Koppold-Liebscher DA, Schuppelius B, Steckhan N, Pfeiffer AFH, Kramer A, et al. Effects of early vs. late time-restricted eating on cardiometabolic health, inflammation, and sleep in overweight and obese women: a study protocol for the ChronoFast trial. Front Nutr. 2021;8:765543. 34869534 10.3389/fnut.2021.765543 PMC8634676

[12] Peters B, Pappe CL, Koppold DA, Schipp K, Arnrich B, Michalsen A, et al. Twenty-four hour glucose profiles and glycemic variability during intermittent religious dry fasting and time-restricted eating in subjects without diabetes: a preliminary study. Nutrients. 2024;16:2663. 39203800 10.3390/nu16162663 PMC11357114

[13] Baum Martinez I, Peters B, Schwarz J, Schuppelius B, Steckhan N, Koppold-Liebscher DA, et al. Validation of a smartphone application for the assessment of dietary compliance in an intermittent fasting trial. Nutrients. 2022;14:3697. 36145073 10.3390/nu14183697 PMC9506329

[14] Hutchinson JW, Alba JW, Eisenstein EM. Heuristics and biases in data-based decision making: effects of experience, training, and graphical data displays. J Mark Res. 2010;47:627–42.

[15] Man CD, Micheletto F, Lv D, Breton M, Kovatchev B, Cobelli C. The UVA/PADOVA type 1 diabetes simulator: new features. J Diab Sci Technol. 2014;8:26–34. 10.1177/1932296813514502 PMC4454102 24876534

[16] Bent B, Henriquez M, Dunn J. Cgmquantify: Python and R Software packages for comprehensive analysis of interstitial glucose and glycemic variability from continuous glucose monitor data. IEEE Open J Eng Med Biol. 2021;2:263–6. 35402978 10.1109/OJEMB.2021.3105816 PMC8901031

[17] Garcia-Gonzalez D, Rivero D, Fernandez-Blanco E, Luaces MR. A public domain dataset for real-life human activity recognition using smartphone sensors. Sensors. 2020;20:2200. 32295028 10.3390/s20082200 PMC7218897

[18] Virtanen P, Gommers R, Oliphant TE, Haberland M, Reddy T, Cournapeau D, et al. SciPy 1.0: fundamental algorithms for scientific computing in Python. Nat Methods. 2020;17:261–72. 32015543 10.1038/s41592-019-0686-2 PMC7056644

[19] Pedregosa F, Varoquaux G, Gramfort A, Michel V, Thirion B, Grisel O, et al. Scikit-learn: machine learning in Python. J Mach Learn Res. 2011;12:2825–30.

[20] Lemaître G, Nogueira F, Aridas CK. Imbalanced-learn: a Python toolbox to tackle the curse of imbalanced datasets in machine learning. J Mach Learn Res. 2017;18:1–5.

[21] Batista GEAPA, Prati RC, Monard MC. A study of the behavior of several methods for balancing machine learning training data. ACM SIGKDD Explor Newsl. 2004;6:20–9.

[22] Chawla NV, Bowyer KW, Hall LO, Kegelmeyer WP. SMOTE: synthetic minority over-sampling technique. J Artif Intell Res. 2002;16:321–57.

[23] Grundler F, Mesnage R, Ruppert PMM, Kouretas D, Wilhelmi De Toledo F. Long-term fasting-induced ketosis in 1610 subjects: metabolic regulation and safety. Nutrients. 2024;16:1849. 38931204 10.3390/nu16121849 PMC11206495

[24] Cartier LJ, Collins C, Lagacé M, Douville P. Comparison of fasting and non-fasting lipid profiles in a large cohort of patients presenting at a community hospital. Clin Biochem. 2018;52:61–6. 29129625 10.1016/j.clinbiochem.2017.11.007

[25] Hall H, Perelman D, Breschi A, Limcaoco P, Kellogg R, McLaughlin T, et al. Glucotypes reveal new patterns of glucose dysregulation. PLoS Biol. 2018;16:e2005143. 30040822 10.1371/journal.pbio.2005143 PMC6057684

[26] Van Doorn WPTM, Foreman YD, Schaper NC, Savelberg HHCM, Koster A, Van Der Kallen CJH, et al. Machine learning-based glucose prediction with use of continuous glucose and physical activity monitoring data: the Maastricht Study. PLoS ONE. 2021;16:e0253125. 34166426 10.1371/journal.pone.0253125 PMC8224858

[27] Bain EE, Shafner L, Walling DP, Othman AA, Chuang-Stein C, Hinkle J, et al. Use of a novel artificial intelligence platform on mobile devices to assess dosing compliance in a phase 2 clinical trial in subjects with schizophrenia. JMIR MHealth UHealth. 2017;5:e18. 28223265 10.2196/mhealth.7030 PMC5340925

