# Supplementary material for: Sensor-based evaluation of intermittent fasting regimes: a machine learning and statistical approach
Source: Int J Obes (Lond). 2025 Aug 22;49(11):2378–82. doi: 10.1038/s41366-025-01889-0 (PMC12583124; doi:10.1038/s41366-025-01889-0)
Supplement: Supplementary file 1 — Table S1: Summary table of the included study. [file 41366_2025_1889_MOESM1_ESM.docx]

**SUPPLEMENTAL**

**Table S1: Summary table of the included study.** Selected glucose features are: mean glucose, mean of Interdaycv (of 45 min feature), mean of J-index (of 45 min feature) and mean of glucose maximum (of 45 min feature). Selected acceleration features are: mean steps, mean vector magnitude, mean of z-axis mean (of 45 min feature), mean of y-axis max (of 45 min feature) and mean of z-axis energy (of 45 min feature).

|  | **Screening N=31**  **mean±sd** | **eTRE N=31**  **mean±sd** | **lTRE N=31**  **mean±sd** |
| --- | --- | --- | --- |
| Average age | 58 (52-65) | | |
| Gender distribution | 100% female | | |
| Average BMI (kg/m^2^) | 30±2.9 | | |
| mean Glucose (mg/dl) | 95.6±6.2 | 85.8±6.3 | 97.4±6.4 |
| mean Steps (count) | 10.8±0.1 | 10.6±0.1 | 10.7±0.1 |
| mean VM (magnitude) | 2380.6±34.2 | 2331.9±34.5 | 2313.8±34.5 |
| mean Interdaycv | 4.6±0.1 | 4.7±0.2 | 5.0±0.1 |
| mean J-index | 11.0±0.6 | 12.1±0.7 | 12.1±0.7 |
| mean Glucose maximum (mg/dl) | 104.0±2.8 | 108.0±2.8 | 109.3±2.8 |
| mean (z-axis mean) | 1562.7±98.1 | 1551.6±99.1 | 1387.3±97.2 |
| mean (y-axis max) | 2346.6±91.9 | 2309.5±91.1 | 2172.2±90.1 |
| mean (z-axis energy) | 152196.0±1214.5 | 151064.8±12001.7 | 129645.7±12705.5 |

**SUPPLEMENTAL B**

We did not have any Type 1 diabetes (T1DM) patients available in our training data due to the absence of T1DM patient cohorts. Therefore, we aimed to apply our algorithm to an unseen test set to evaluate how well it generalizes, even for individuals with T1DM.

To achieve this, we utilized Simglucose, a Python package designed for simulating T1DM time series data. Simglucose allows for various configurations, such as selecting the age of simulated individuals and the glucose measurement device. Since managing T1DM requires insulin administration, the simulation incorporates insulin delivery via an insulin pump following each meal.

For this investigation, we configured the simulation as follows:

• Data Generation: 24-hour time series for 10 simulated adults

• Meal Schedule & Intake:

• Breakfast at 8 a.m. (400 grams of food)

• Lunch at 12 p.m. (450 grams of food)

• Dinner at 6 p.m. (550 grams of food)

• No snacks included

• Glucose Measurement: Dexcom CGM sensor

• Insulin Delivery: Deltec Cozmo insulin pump with a Basal-Bolus controller

• Sampling Rate: 1 sample every 3 minutes, using continuous glucose monitoring (CGM) in mg/dL

• Fasting States: Defined based on meal times, considering a postprandial glucose shift of four hours

By incorporating this simulated dataset, we could assess the model’s performance on previously unseen T1DM data, helping to determine its generalizability beyond the original training population.

**SUPPLEMENTAL C**

Dashboard for fasting state prediction using a support vector machine as the machine learning predictor. The background color represents the predicted fasting states, with red indicating fasting and blue indicating non-fasting. The dots represent glucose values labeled as fasting or non-fasting according to the individual’s dietary protocol. The compliance score for a participant (not part of the training data set) in the ChronoFast study is 86%.
